# The role of routine FIBERoptic bronchoscopy monitoring during percutaneous dilatational TRACHeostomy (FIBERTRACH): a study protocol for a randomized, controlled clinical trial

**DOI:** 10.1186/s13063-021-05370-x

**Published:** 2021-06-29

**Authors:** José M. Añón, María Soledad Arellano, Manuel Pérez-Márquez, Claudia Díaz-Alvariño, José A. Márquez-Alonso, Jorge Rodríguez-Peláez, Kapil Nanwani-Nanwani, Ana Martín-Pellicer, Belén Civantos, Alba López-Fernández, Irene Seises, Jorge García-Nerín, Juan C. Figueira, Henar Casero, Javier Vejo, Alexander Agrifoglio, Lucía Cachafeiro, Mariana Díaz-Almirón, Jesús Villar

**Affiliations:** 1 Intensive Care Unit, Hospital Universitario La Paz, Paseo de la Castellana 261, 28046 Madrid, Spain; 2 Instituto de Investigación del Hospital Universitario La Paz (IdiPAZ), Madrid, Spain; 3 CIBER de Enfermedades Respiratorias, Instituto de Salud Carlos III, Madrid, Spain; 4 Intensive Care Unit, Hospital Universitario Rey Juan Carlos, Madrid, Spain; 5 Biostatistics Research Unit, Hospital Universitario La Paz, Madrid, Spain; 6 Research Unit, Hospital Universitario Dr. Negrín, Las Palmas de Gran Canaria, Spain; 7 Keenan Research Center for Biomedical Research, Li Ka Shing Knowledge Institute, St. Michael’s Hospital, Toronto, Canada

**Keywords:** Percutaneous tracheostomy, Endoscopic guide, Fiberoptic bronchoscopy, Complications, Prolonged mechanical ventilation

## Abstract

**Background:**

Tracheostomy is one of the most frequent techniques in intensive care units (ICU). Fiberoptic bronchoscopy (FB) is a safety measure when performing a percutaneous dilatational tracheostomy (PDT), but the controversy surrounding the routine use of FB as part of the procedure remains open. National surveys in some European countries showed that the use of FB is non-standardized. Retrospective studies have not shown a significant difference in complications between procedures performed with or without a bronchoscope. International guidelines have not been able to establish recommendations regarding the use of FB in PDT due to lack of evidence.

**Design:**

This is a multicenter (three centers at the time of  publishing this paper) randomized controlled clinical trial to examine the safety of percutaneous tracheostomy using FB. We will include all consecutive adult patients admitted to the ICU in whom percutaneous tracheostomy for prolonged mechanical ventilation is indicated and with no exclusion criteria for using FB. Eligible patients will be randomly assigned to receive blind PDT or PDT under endoscopic guidance. All procedures will be performed by experienced intensivists in PDT and FB. A Data Safety and Monitoring Board (DSMB) will monitor the trial. The primary outcome is the incidence of perioperative complications.

**Discussion:**

FB is a safe technique when performing PDT although its use is not universally accepted in all ICUs as a routine practice. Should PDT be monitored routinely with endoscopic guidance? This study will assess the role of FB monitoring during PDT.

**Trial registration:**

ClinicalTrials.gov NCT04265625. Registered on February 11, 2020

**Supplementary Information:**

The online version contains supplementary material available at 10.1186/s13063-021-05370-x.

## Background

Tracheostomy is one of the most frequently performed techniques in intensive care units (ICUs). The era of modern tracheostomy has a starting point in 1909 when Chevalier Jackson [1] describes the surgical tracheostomy. In 1985, Ciaglia et al. [2] described the percutaneous dilatational tracheostomy (PDT) that met the requirements of simplicity, swiftness, and safety, which, along with the possibility of being performed at the bedside, developed a new way of understanding surgical airway access in the critically ill patient. Subsequently, changes [3–5] over the classical technique and other modalities were developed [6–8].

A few years after the description of PDT, Paul et al. [9] performed tracheostomies using endoscopic guidance in four patients, on the basis that the blind nature of PDT justified most of the complications associated with it. The duration of the procedure in all four patients lasted between 10 and 15 min, and the authors stated that the use of endoscopic guidance prevented damage to the posterior tracheal wall and helped in identifying the puncture site thanks to transillumination. Currently, the “routine” use of endoscopic guidance as part of percutaneous tracheostomy is controversial [10–13] and guidelines cannot establish recommendation levels [14–16].

We postulate that percutaneous tracheostomy performed under endoscopic control does not reduce the incidence of perioperative complications of the procedure in critically ill patients when it is performed by experienced physicians in patients without anatomical abnormalities.

## Methods/design

### Justification of the study

Besides a few recommendations that have been made regarding some modalities [4, 5, 8], endoscopic guide as part of PDT is a controversial issue. Some authors suggest that it is a safety measure that can prevent serious complications and should be considered in all PDT procedures in the absence of contraindications [17–19]. For others [10], these studies are limited and unclear whether visualization of the interior of the airway is a necessary component of the procedure. In two comparative retrospective studies [10, 11], the addition of bronchoscopy was not accompanied by a significant difference in complications between procedures performed with and without a bronchoscope. In a retrospective analysis [12] involving trauma patients, the use of fiberoptic bronchoscopy (FB) when performing PDT was not routinely required but might be used as an important adjunct in selected patients. In a retrospective review [13] of 3162 PDT, the largest reported series percutaneous tracheostomy in critically ill patients, the authors conclude that routine bronchoscopic guidance is not necessary.

The advantages of using a FB are the ability to transilluminate the neck and directly visualize the trachea during needle insertion and dilation. In patients with difficult airways or altered anatomy, the bronchoscope allows for repositioning of the needle to avoid puncturing surrounding structures [20]. FB is a safety measure when performing PDT, although its use in mechanically ventilated patients could be associated with some risks (i.e., auto-PEEP [21], significant hypercarbia [22]). In addition, the bronchoscope may increase the procedure time and costs [10].

It has been suggested [23, 24] that bronchoscopy should be used by surgeons who are less skilled with PDT, as a method to reduce complications or in case of anatomical challenge [25]. FEPIMCTI [14] guidelines do not establish recommendations regarding this technique because there is insufficient evidence to suggest that using a bronchoscope while performing PDT reduces the rate of complications. The French Society of Intensive Care [15] guidelines established a weak recommendation based on a study in 60 patients in which endoscopic guidance was associated with fewer minor complications. The Indian Society of Critical Care Medicine expert panel states that fiberoptic bronchoscope may be used, whenever available to aid PDT although it does not reduce the rate of complications [16].

Currently, evidence regarding the use of FB in PDT is practically non-existent. Large prospective randomized studies are needed comparing PDT with and without FB, performed by experienced personnel in both the PDT technique and the endoscopic guidance. This rationale is the basis and justification for the present study.

The aim of this study is to assess the occurrence of perioperative complications associated with the procedure of percutaneous tracheostomy under endoscopic guidance versus blind percutaneous tracheostomy.

### Study design

#### Design

This is a randomized, controlled, multicenter clinical trial (three centers at the time of publishing this paper, Hospital Universitario La Paz, Hospital Universitario Rey Juan Carlos and Hospital Universitario Infanta Leonor in Madrid, Spain). The trial was designed in accordance with the Declaration of Helsinki, the Convention of the European Council related to human rights and biomedicine, and within the requirements established by Spanish legislation in the field of biomedical research, the protection of personal data and bioethics. The trial was registered on the 11th of February 2020 at http://www.clinicaltrials.gov with identification no. NCT 04265625. The study protocol (Version 1, 11th October 2019) was approved by the Ethics Committee (Hospital Universitario La Paz, Madrid, Spain, identification no. 5455 -Additional file 1) and by the Institutional Review Board of Hospital Universitario Rey Juan Carlos and Hospital Universitario Infanta Leonor, Madrid, Spain. For inclusion into the study, approval on a written informed consent will be requested by the local investigators from the patient’s relatives or legal representatives (Additional file 2). Our protocol followed the SPIRIT (Standard Protocol Items: Recommendations for Interventional Trials) guidelines. See Additional file 3 for the SPIRIT checklist of the study protocol.

#### Study population

We will include patients admitted to the ICU requiring percutaneous tracheostomy for prolonged mechanical ventilation. Inclusion criteria are (i) hospitalized adults of ≥ 18 years at the time of screening, and (ii) subjects (or a legal representative, or the nearest relative, or a relative by marriage, as appropriate) provide signed informed consent prior to initiation of any study procedures. We will exclude the following patients: (i) patients with increased intracranial pressure (ICP), either suspected or observed by its monitoring; (ii) patients who have any absolute or relative contraindications [26] to the performance of PDT: morbid obesity, neck deformity, coagulopathy (INR> 1.5) or thrombopenia (platelet count <50,000) with difficulty in reversal, need to establish an emergency surgical airway, cervical spine injury, previous neck surgery or previous tracheostomy, surgical area infection and high oxygen requirements (FiO_2_ ≥ 0.7), and PEEP (> 10 cmH_2_O); and (iii) patients in whom, for safety reasons, the physician in charge considers that the procedure should be monitored with endoscopic guidance.

#### PDT modality used

PDT will be performed with the single dilatation method, as it is the usual practice in the ICU in La Paz University Hospital. For both arms, the patient will be placed with the neck extended and the usual aseptic measures will be taken in the surgical area. All patients will be monitored with electrocardiographic control, invasive blood pressure, and continuous pulse oximetry. A FiO_2_ of 100% will be maintained throughout the procedure with correct visualization of the curves and parameters obtained from the mechanical ventilator. Sedation and analgesia will be initiated, and if it is already being infused continuously due to its basal situation, an extra dose will be administered if necessary along with a bolus dose of a non-depolarising neuromuscular blocking agent (usually cisatracurium).

#### Randomization

Candidate patients will be randomized with a 1:1 ratio. Randomization will be performed with the sealed envelope system. Patients randomized to the “PDT with endoscopic guidance” arm will undergo the procedure controlled with a fiberoptic bronchoscope (as is usual clinical practice when dealing with patients with anatomical challenges or difficult airways). The bronchoscope will be introduced through the endotracheal tube (ETT), and under fibroscopic vision, ETT will be placed in such a way that the balloon is just below the vocal cords. To minimize the adverse effects that can be derived from the use of the fiberoptic bronchoscope, the difference between the internal diameter of the ETT and the external diameter of the bronchoscope will be at least 2 mm [21]. In patients randomized to the “PDT without endoscopic guidance” group, ETT will be placed with the balloon under the vocal cords under direct laryngoscopy vision, as it is the usual practice when dealing with patients without difficult airway or anatomical challenge.

#### Percutaneous tracheostomy technique with a single dilator

Once the patient and the surgical field are prepared, anatomical structures (cricoid cartilage, cricothyroid membrane, and tracheal rings) are identified by palpation, and subsequently, an incision (preferably horizontal) is made in the skin of approximately 1.5–2 cm above the point where the tracheal puncture will be performed, followed by a blunt dissection of soft tissues in order to easily locate the tracheal structures. A tracheal puncture will be performed via the incision, preferably between the first and second tracheal ring. After confirming the location in the tracheal lumen by air aspiration (or direct vision with fiberoptic bronchoscope in patients randomized to this arm), the needle and syringe are withdrawn, leaving the tracheal sheath in the tracheal lumen through which the J-shaped metal guide will be inserted. Subsequently, the sheath is removed and the first dilatation is carried out through the metal guide with the small 14Fr dilator. Then, definitive dilatation will be performed with the Teflon catheter that has been previously built into the curved dilator with the hydrophilic covering. This curved dilator has to be moistened with physiological saline solution prior to its introduction to soften the structure. Subsequently, it is introduced through the metal guide through the soft tissues into the tracheal lumen (up to the external signal of 38Fr). Once this maximum, dilation has been achieved, the curved dilator is extracted, and the tracheostomy cannula along with the cannula introducer is advanced over the metal guide and the Teflon catheter into the tracheal lumen. Once the tracheostomy cannula has been introduced, the cannula introducer, the Teflon catheter, and the metal guide are removed (along with the fiberoptic bronchoscope in patients randomized to this arm). Finally, secretions are aspirated, the tracheal cannula balloon is inflated, and the patient is connected to the ventilator.

#### Level of experience of the personnel involved in the technique

Percutaneous tracheostomy has been performed at the coordinator center since 1991 [27], and during many years, it was done without bronchoscopy. Over time, with the addition of other faculty, and as an adjunct in residency training, the use of FB was added to the procedure (although no routinely). Physicians of participating centers are highly experienced in PT and the use of FB. Therefore, the procedure will be performed by experienced personnel and those procedures performed by physicians in the learning curve (i.e., residents), it will be directly supervised by experienced intensivists [5].

#### Data collection

In addition to demographic variables, and duration of the procedure (from skin incision to placement of tracheostomy tube), we will collect perioperative complications (following the classification proposed by Durbin [28] modified [29]. Minor complications include those that are easily corrected, with no risk of sequelae or need of unscheduled therapeutic strategies for their solution: (a) haemorrhage: bleeding controlled with digital pressure that does not cause hemodynamic instability, nor requires surgical revision or transfusion; (b) transient desaturation: desaturation during the procedure (SpO_2_<90% but ≥ 85%); (c) loss of airway control: failure to secure the airway without impact on SpO_2_ (not <90%); (d) atelectasis: partial atelectasis (segmental or lobar) without a decrease in SpO_2_<90% and that does not require fiberoptic bronchoscopy for its resolution; (e) arterial hypotension, which requires expansion with less than 1000 ml of fluids and does not require administration or increase of previous doses of inotropics; (f) barotrauma: subcutaneous emphysema; (g) rupture of tracheal rings: rupture of the tracheal ring during any phase of the procedure (only in patients in whom endoscopic guidance is performed); and (h) technical problems without clinical repercussions: puncture of the ETT cuff, difficulty in inserting the cannula or impossibility to complete the technique as an isolated complication that did not lead to repercussions such as desaturation or loss of the airway. Major complications include those when an unscheduled therapeutic act was required, or when the complication entails potential risk (although there had been no life threat or consequential sequelae), or there is a real risk to life, cardiac arrest, or death directly related to the complication derived from the technique: (a) hemorrhage: bleeding that causes hemodynamic instability, and/or requires surgical revision, and/or transfusion; (b) desaturation (SpO_2_<85%) of any duration; (c) loss of airway control causing a drop in SpO_2_<85%; (d) total or partial atelectasis that have an impact on SpO_2_ or require a therapeutic maneuver for its solution (fiberoptic bronchoscopy); (e) hypotension that requires treatment with more than 1000 cc of fluids, and/or initiation of vasopressors, or an increase in previous doses; (f) false passage that causes injury to the trachea or mediastinal emphysema or desaturation (SpO_2_ <85%); (g) barotrauma: pneumothorax or mediastinal emphysema; (h) rupture or tear of the tracheal wall; (i) injury to the posterior wall of the trachea: damage produced to the posterior wall of the trachea with the needle, guide, or dilator; and (j) technical problems with clinical repercussions: puncture of the ETT cuff, difficulty in inserting the cannula, or impossibility of completion of the technique that entails consequences such as desaturation or loss of the airway or serious complications that require a change in strategy.

We will also collect the arterial partial pressure of CO_2_ at the beginning and end of the procedure, the arterial partial pressure of O_2_ at the beginning and end of the procedure, pH at the beginning and end of the procedure, minimum arterial oxygen saturation (measured by pulse oximetry) during the procedure, and maximum end-tidal CO_2_ during the procedure (if capnography is available).

#### Statistical analysis plan

Data will be collected in each participating ICU using a standardized form. Then, data will be transmitted to the coordinating center whenever a patient dies or is discharged from the hospital. Before exporting the data into a computerized database at the randomization center, the coordinator (JMA) will check the completeness and the quality of information. Logical checks will be performed for missing data and to find inconsistencies. If necessary, the data collector will contact the investigator by phone to validate the data or reformat the data for entry into the database. Considering that the rate of complications described in published work in which PDT with FB was performed ranged between 3.7% and 16%, and those without FB (eliminating extreme values) ranged between 11% and 25%, for a confidence level of 95%, a statistical power of 80% and a potential loss of 10%, a sample size of 221 patients has been calculated for each arm. Collected data will be anonymized in compliance with the Spanish Law 3/2018, of December 5, on Data Protection and Guarantee of Digital Rights. The results of the quantitative variables will be expressed as mean and standard deviation when they fit a normal distribution curve evaluated with the test of Kolmogorov-Smirnoff. Data without a normal distribution will be expressed as median and interquartile range. Qualitative data will be expressed through its absolute frequency and percentage. The comparison between quantitative variables will be carried out using the Student’s t test or the Mann-Whitney test, depending on the distributions of the variables analyzed. The chi-squared test will be used to demonstrate differences between qualitative variables. No imputation will be made of the lost data. All tests will be considered two-tailed at a 95% level of significance. All statistical analyses will be performed using statistical software SAS Enterprise Guide 8.2 (Cary NC, SAS Institute Inc., USA). Statistical significance will be set at p<0.05.

#### Trial organization

The steering committee is composed of the study’s principal investigators who contributed to its design and approved the final protocol (Appendix 1). The trial will be monitored by a Data Safety and Monitoring Board (DSMB) (Appendix 2). The DSMB can recommend stopping the trial due to safety concerns. The DSMB will be composed of three independent experts in critical care medicine (n=2) and surgery (n=1). All severe complications will be reported to the institutional review board and DSMB within 24 h after the procedure. The study coordinator (JMA) is responsible for promoting patient enrolment and follow-up, including a list of any outcome data to be collected from participants who discontinue or deviate from intervention protocols. Other responsibilities of the study coordinator and the trial management team, where a statistician is included, are (i) planning and conducting the study: designing the protocol, designing the randomization process, and managing and controlling the data quality; (ii) monitoring recruitment rates, taking actions to increase patient enrolment, and taking actions to increase the number of participating centers; and monitoring follow-up and auditing (iii) statistical analysis, research reporting, and helping in writing the final manuscript.

## Discussion

This is a protocol for a randomized controlled trial designed to evaluate the efficacy of percutaneous tracheostomy under endoscopic guidance versus blind percutaneous tracheostomy (always performed by experienced intensivists) and to evaluate the effects on ventilatory parameters during percutaneous tracheostomy with and without endoscopic control.

National surveys carried out in some European countries showed that the use of FB as part of the technique is used in a heterogeneous way, ranging between 16% and 98%, although the “time” factor in which each of them was carried out has to be taken into account [30–35].

In the earlier studies [17–19], it was found that bronchoscopic guidance reduces the risk of complications and is safe and cost-effective, but the recent comparative studies (although retrospective) have suggested that PDT can be safely performed without bronchoscopic guidance without any increase in complications and it is not mandatory to do bronchoscopy guided PDT [10–13]. Some authors have even hypothesized discarding the use of bronchoscopy for this procedure in order to potentially decrease its cost and increase its efficiency with similar outcomes compared with bronchoscopy-assisted PDT [10]. International guidelines [14] have not been able to establish a recommendation regarding this measure due to a lack of evidence. Therefore, the controversy surrounding the routine use of FB as part of PDT remains open.

The main difference between our study and other studies is that our study is the first prospective randomized clinical trial to assess this topic. All results about this topic are based on retrospective studies or case series. Should PDT be monitored routinely with endoscopic guidance? So far, there is no evidence to provide a definitive answer.

### Trial status

The first patient was enrolled on December 7, 2019. The expected duration of the study is 3 years.

### Supplementary Information

**Additional file 1.** Approval of the Ethics Committee (according to the Spanish legislation #RD 1090/2015, this approval is mandatory for all participating centers).

**Additional file 2.** Informed consent form.

**Additional file 3.** SPIRIT checklist.

## Data Availability

JMA, MSA, CDA, JCF, BC, AA, LC, MDA, and JV will have full access to all data at the end of the study and take responsibility for the integrity of the data and the accuracy of the data analysis. All data needed to evaluate the conclusions of the trial will be present and tabulated in the main text or in the supplementary material of the final publication. For individual de-identified raw data will be available from the corresponding author on reasonable request.

## Appendix 1

Steering Committee: José M. Añón, María Soledad Arellano, Claudia Díaz-Alvariño, Alexander Agrifoglio, Lucía Cachafeiro, Jesús Villar.

## Appendix 2

(DSMB): Gonzalo Hernández (Intensive Care Unit, Hospital Virgen de la Salud, Toledo, Spain). Federico Gordo (Intensive Care Unit, Hospital Universitario del Henares, Madrid, Spain). José Luis Cebrián (Department of Oral and Maxillofacial Surgery, Hospital Universitario La Paz, Madrid, Spain).

## Appendix 3

FIBERTRACH investigators: José M. Añón, María Soledad Arellano, Claudia Díaz-Alvariño, Jorge Rodríguez-Peláez, Kapil Nanwani, Belen Civantos, Alba López-Fernández, Irene Seises, Jorge García Nerín, Juan C. Figueira, Henar Casero, Javier Vejo, Alexander Agrifoglio, Lucía Cachafeiro, Mariana Díaz-Almirón (Hospital Universitario La Paz, Madrid, Spain). Manuel Pérez Márquez, José A. Márquez-Alonso, Ana Martín-Pellicer (Hospital Universitario Rey Juan Carlos, Madrid, Spain), Covadonga Rodríguez, Renata García, María Paz Escuela (Hospital Universitario Infanta Leonor, Madrid, Spain)
