# Supplementary material for: The role of routine FIBERoptic bronchoscopy monitoring during percutaneous dilatational TRACHeostomy (FIBERTRACH): a study protocol for a randomized, controlled clinical trial
Source: Trials. 2021 Jun 29;22:423. doi: 10.1186/s13063-021-05370-x (PMC8240418; doi:10.1186/s13063-021-05370-x)
Supplement: Supplementary file 1 — Additional file 1. Approval of the Ethics Committee (according to the Spanish legislation #RD 1090/2015, this approval is mandatory for all participating centers). [file 13063_2021_5370_MOESM1_ESM.pdf]

Hospital Universitario La Paz
Hospital Carlos III
Hospital Cantoblanco
SaludMadrid

REPORT OF THE CLINICAL RESEARCH ETHICS COMMITTEE WITH MEDICINAL PRODUCTS

Mrs Almudena Castro Conde, Chairman of the Clinical Research Ethics Committee with medicinal products of Hospital Universitario La Paz

CERTIFIES

That this Committee has evaluated the proposal made by Dr. José Manuel Añón Elizalde, of Hospital Universitario "La Paz" to perform the study entitled "**ROUTINE USE OF FIBEROPTIC BRONCHOSCOPIC GUIDANCE IN PERCUTANEOUS TRACHEOSTOMY (FIBERTRACH)",** Version 1.0 of October 11, 2019 and Legal representative/family Information Sheet and Informed Consent Version 1.0 of October 2019, HULP code: **5455**

and considers that taking into account the response to the clarifications requested,

- The necessary requirements for the suitability of the protocol in relation to the objectives of the study are met and the foreseeable risks and inconveniences for the subject are justified.
- The investigator's capacity and available resources are appropriate to carry out the study.
- The procedure for obtaining informed consent is appropriate and the scope of the expected financial compensation does not interfere with respect for ethical postulates.
- The scope of the financial compensation planned does not interfere with respect tothe ethic postulates.

That this Committee accepts this study be carried out by the researcherJosé Manuel Añón Elizalde, of the Intensive Medicine Department of Hospital Universitario "La Paz", as principal investigator.

What I sign in Madrid on Octuber 24, 2019

Signed:
Dr. Almudena Castro Conde

Firmado digitalmente por: FERNANDEZ DE UZQUIANO MARIA EMMA
Fecha: 2021.04.26 09:24

Dr. Emma Fernandez de Uzquiano
Secretary of the Clinical Research Ethics Committee
On behalf of

La autenticidad de este documento se puede comprobar en www.madrid.org/csv
mediante el siguiente código seguro de verificación: 1212479236710472836 57
