# Supplementary material for: The role of routine FIBERoptic bronchoscopy monitoring during percutaneous dilatational TRACHeostomy (FIBERTRACH): a study protocol for a randomized, controlled clinical trial
Source: Trials. 2021 Jun 29;22:423. doi: 10.1186/s13063-021-05370-x (PMC8240418; doi:10.1186/s13063-021-05370-x)
Supplement: Supplementary file 2 — Additional file 2. Informed consent form. [file 13063_2021_5370_MOESM2_ESM.doc]

**HOJA DE INFORMACIÓN AL FAMILIAR O REPRESENTANTE LEGAL**

**Titulo:** Papel del uso rutinario de la guía endoscópica en la traqueotomía percutánea por dilatación. Estudio prospectivo multicéntrico y aleatorizado

**Investigador Principal, servicio/unidad y centro:** José Manuel Añón Elizalde. Servicio de Medicina Intensiva. Hospital Universitario La Paz

**Introducción**

Nos dirigimos a usted para informarle sobre un estudio en el que se le invita a participar a su familiar. Nuestra intención es que reciba la información correcta y suficiente para que pueda evaluar y juzgar si quiere o no participar en este estudio. Nosotros le aclararemos las dudas que puedan surgir en cualquier momento. Además, puede consultar con las personas que considere oportuno.

**Participación voluntaria**

Debe saber que la participación es voluntaria y que puede decidir no participar o cambiar su decisión y retirar el consentimiento en cualquier momento, sin que por ello se altere la relación con su médico ni se produzca perjuicio alguno en su tratamiento.

**Descripción y objetivo general del estudio**

Su familiar está ingresado en el Servicio de Medicina Intensiva y va a precisar ventilación mecánica durante más de 10 días. Por ello y siguiendo la práctica clínica habitual es necesario practicarle una traqueotomía. La modalidad de traqueotomía a realizar en nuestro Servicio y en la gran mayoría de los Servicios de Medicina Intensiva es la denominada traqueotomía percutánea. Se trata de un procedimiento a realizar a pie de cama (en el mismo Servicio de Medicina Intensiva sin necesidad de traslado a quirófano) y se denomina “percutánea” porque a diferencia de la traqueotomía quirúrgica se realiza mediante un sistema de dilatación sin necesidad de la apertura quirúrgica por planos hasta llegar a la tráquea. Este sistema de dilatación permite la realización de un orificio en la traquea (denominado traqueostoma) a través del que se inserta una cánula de traqueotomía para que el paciente pueda ser ventilado a través de ésta en lugar de ser ventilado por el tubo que en el momento actual tiene introducido en de la boca. Desde hace años se discute si este tipo de traqueotomía debe realizarse con control endoscópico que significa que la técnica será monitorizada por un fibrobroncoscopio que se introduce a través del tubo orotraqueal (durante el procedimiento de la traqueotomía) o debe realizarse sin este control. La discusión se basa en que el control endoscópico puede prevenir complicaciones pero se invierte más tiempo y puede haber eventos adversos si no se tiene experiencia o no se utiliza con las precauciones propias de esta técnica en el paciente ventilado. La variabilidad en cuanto al uso se conoce por encuestas nacionales publicadas tanto en nuestro país como en países de nuestro entorno. En países como Alemania e Italia se utiliza en el 98% y 93% de los Servicios de Medicina Intensiva. En nuestro país en una encuesta publicada en 2004 se utilizaba como rutina en el 16% de ellas.

Por ello estamos realizando un estudio que tiene por objeto conocer si la utilización de la fibrobroncoscopia como parte del procedimiento de la traqueotomía percutánea aporta beneficios sobre la realización de la traqueotomía percutánea sin su utilización. Para ello se van a establecer dos grupos de pacientes. A un grupo se le realizará traqueotomía percutánea sin guía endoscópica y a otro grupo se le realizará con guía endoscópica. El estudio es aleatorizado. Esto significa que pertenecer a un grupo o a otro va a ser al azar y la probabilidad de estar en uno u otro grupo será del 50%.

**Riesgos y molestias derivados de su participación en el estudio**

La participación en el estudio no entraña riesgo o molestia adicional para el paciente. La traqueotomía percutánea es práctica habitual en los pacientes ventilados mecánicamente de forma prolongada y el Servicio de Medicina Intensiva del Hospital Universitario La Paz cuenta con una amplia experiencia. Se realizan aproximadamente 80-100 procedimientos anuales. La utilización de la guía endoscópica se realiza también como práctica habitual en aquellos pacientes cuya anatomía podría ofrecer dificultad para el desarrollo de la traqueotomía.

La participación en el estudio no conlleva visitas posteriores ni cuestionarios ni procedimientos adicionales puesto que tan solo se tomarán los datos relacionados con la técnica en el momento de su ejecución.

**Posibles beneficios**

La participación en el estudio no aporta ningún beneficio para el paciente. Contribuirá a favorecer el conocimiento en el campo de la traqueotomía en los pacientes ingresados en las UCI a los que se precisa realizar esta técnica.

**Compensación económica**

Su participación en el estudio no le supondrá ningún gasto adicional ni tendrá compensación económica alguna.

**Contacto**

Si tuviera alguna pregunta en el futuro sobre la revelación o el uso que se pudiera hacer de sus datos médicos, si tuviera dudas, preocupaciones o quejas sobre el estudio o su participación en él, deberá contactar con:

• Dr José Manuel Añón Elizalde en el teléfono …646823223

# CONSENTIMIENTO INFORMADO

**Titulo:** Papel del uso rutinario de la guía endoscópica en la traqueotomía percutánea por dilatación. Estudio prospectivo multicéntrico y aleatorizado

**Investigador Principal:** Dr. José Manuel Añón Elizalde, Médico Adjunto del Servicio de Medicina Intensiva, Hospital universitario La Paz

# Consentimiento

Yo (nombre y apellido)___________________________________en mi propio nombre y representación,

o en nombre y representación de D./Dña. ____________________________________, (se debe acreditar tal condición)

- He leído la hoja de información que se me ha entregado y he podido hacer preguntas y recibido suficiente información sobre el estudio. Además comprendo que puedo retirarme del estudio cuando quiera, sin tener que dar explicaciones y sin que esto repercuta en mis cuidados médicos.
- Al facilitar mis datos garantizo haber leído y aceptado expresamente el tratamiento de los mismos conforme a lo indicado.
- Presto libremente mi conformidad para participar en el estudio.

Y para que así conste lo firmó en Madrid a_____ de_________________ 20___.

Firma:........................................ Firma:.........................................

Investigador: __________________ Paciente o familiar_______________

**CONFIDENCIALIDAD/PROTECCIÓN DE DATOS**

**CONSENTIMIENTO PARA ESTUDIOS DE INVESTIGACIÓN**

Mediante el presente escrito y en cumplimiento de la normativa vigente en materia de protección de datos, quedo informado/a y consiento expresamente el tratamiento de los datos de mi historia clínica así como los resultantes de su participación en el estudio Papel del uso rutinario de la guía endoscópica en la traqueotomía percutánea por dilatación. Estudio prospectivo multicéntrico y aleatorizado. El Responsable del Tratamiento es Hospital Universitario La Paz (incluido Hospital Carlos III-Hospital Cantoblanco) cuyo Delegado de Protección de Datos (DPD) es el **“***Comité PDP de la Consejeria de Sanidad de la Comunidad de Madrid”* con dirección en Plaza Carlos Trías Bertrán nº7 (Edificio Soluble) Madrid 28020 [protecciondedatos.sanidad@madrid.org](mailto:protecciondedatos.sanidad@madrid.org). La finalidad es conocer si la utilización de la fibrobroncoscopia como parte del procedimiento de la traqueotomía percutánea aporta beneficios sobre la realización de la traqueotomía percutánea sin su utilización

La base jurídica que legitima el tratamiento es su consentimiento, así como la *Ley 14/2007, de 3 de julio, de Investigación biomédica* y demás legislación vigente en la materia. Con esta finalidad sus datos serán conservados durante los años necesarios para cumplir con las obligaciones estipuladas en la normativa vigente aplicable, así como mientras que sea de utilidad para la finalidad para la que fue obtenida, y en cualquier caso, al menos durante cinco años. El acceso a mi información personal quedará restringido al médico/s del estudio, sus colaboradores y demás personal que participe en el mismo, autoridades sanitarias, Comité Ético de Investigación del Hospital y a los monitores y auditores del promotor, quienes estarán sometidos al deber de secreto inherente a su profesión, cuando lo precisen, para comprobar los datos y procedimientos del estudio, pero siempre manteniendo la confidencialidad de los mismos de acuerdo a la legislación vigente. No se realizarán comunicaciones adicionales de datos, salvo en aquellos casos obligados por Ley.

Al facilitar sus datos usted garantiza haber leído y aceptado expresamente el tratamiento de los mismos conforme a lo indicado. Podrá ejercer sus derechos de acceso, rectificación, supresión, oposición, limitación del tratamiento y portabilidad**,** en la medida que sean aplicables, a través de comunicación escrita al Responsable del Tratamiento de Datos, con domicilio en Hospital Universitario La Paz, *Paseo de la Castellana 261, 28046 Madrid*, concretando su solicitud, junto con su DNI o documento equivalente. Asimismo, le informamos de la posibilidad de presentar una reclamación ante la Agencia Española de Protección de Datos *(C/Jorge Juan, 6 Madrid 28001)* [www.agpd.es](http://www.agpd.es/).

Y para que así conste lo firmó en Madrid a_____ de_________________ 20___.

**FIRMADO:**

D./Dña. ________________________________________________________________,

con N.I.F. _________________________, en mi propio nombre y representación,

o en nombre y representación de D./Dña. ____________________________________,

con N.I.F. _____________ (se debe acreditar tal condición)
