# Supplementary material for: The role of routine FIBERoptic bronchoscopy monitoring during percutaneous dilatational TRACHeostomy (FIBERTRACH): a study protocol for a randomized, controlled clinical trial
Source: Trials. 2021 Jun 29;22:423. doi: 10.1186/s13063-021-05370-x (PMC8240418; doi:10.1186/s13063-021-05370-x)
Supplement: Supplementary file 3 — Additional file 3. SPIRIT checklist. [file 13063_2021_5370_MOESM3_ESM.doc]

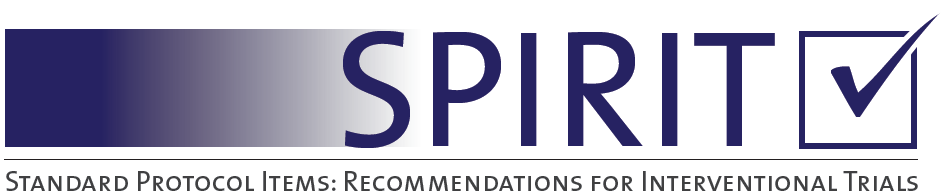


SPIRIT 2013 Checklist: Recommended items to address in a clinical trial protocol and related documents*

| Section/item | ItemNo | Description |
| --- | --- | --- |
| **Administrative information** | | |
| Title | 1 | The role of routine FIBERoptic bronchoscopy monitoring during percutaneous dilatational TRACHeostomy (FIBERTRACH). A study protocol for a randomized, controlled clinical trial (Page 1 of the manuscript) |
| Trial registration | 2a | Registered on ClinicalTrials.gov, number NCT04265625 on 11th February 2020 as FIBERTRACH. (Pag 2 and Pag 5 of the manuscript) |
|  | 2b | The study was not registered at the WHO registration portal. |
| Protocol version | 3 | Version 1, 11th October 2019. Ethical approval #5455. (Pag 5 of the manuscript) |
| Funding | 4 | None (Pag 13 of the manuscript) |
| Roles and responsibilities | 5a | Names, affiliations, and roles of protocol contributors (Pag 12-13 of the manuscript) |
|  | 5b | This is an investigator-initiated, academic, non-industry sponsored trial. |
|  | 5c | This study has not sponsor and funders (Pag 13 of the manuscript) |
|  | 5d | Composition, roles, and responsibilities of the coordinating centre, steering committee, data management team, and other individuals or groups overseeing the trial: Pag 12 and 13 of the manuscript |
| Introduction |  |  |
| Background and rationale | 6a | Pag 3 of the manuscript. |
|  | 6b | Pag 3 of the manuscript. |
| Objectives | 7 | Pag 5 of the manuscript. |
| Trial design | 8 | Pag 5 of the manuscript. |
| Methods: Participants, interventions, and outcomes | | |
| Study setting | 9 | Pag 5 of the manuscript |
| Eligibility criteria | 10 | Pag 5 of the manuscript. |
| Interventions | 11a | Pag 6-7 of the manuscript. |
|  | 11b | Criteria for discontinuing or modifying allocated interventions for a given trial participant (eg, drug dose change in response to harms, participant request, or improving/worsening disease). Not applicable |
|  | 11c | Pag 10 of the manuscript |
|  | 11d | There are no specific concomitant care and interventions that are permitted or prohibited that are applicable to this trial (out of exclusion criteria to be included in the trial) |
| Outcomes | 12 | Pag 8 and 9 of the manuscript |
| Participant timeline | 13 | Not applicable. There is not a follow-up of patients in this trial after ICU discharge |
| Sample size | 14 | Pag 9 of the manuscript |
| Recruitment | 15 | Strategies for achieving adequate participant enrolment to reach target sample size; No applicable |
| **Methods: Assignment of interventions (for controlled trials)** | | |
| Allocation: |  |  |
| Sequence generation | 16a | Pag 6 of the manuscript |
| Allocation concealment mechanism | 16b | Pag 6 of the manuscript |
| Implementation | 16c | Pag 6-7 of the manuscript |
| Blinding (masking) | 17a | No applicable. The trial is not blinded |
|  | 17b | No applicable |
| **Methods: Data collection, management, and analysis** | | |
| Data collection methods | 18a | Data will be collected in each participating ICU using a standardized case report form (CRF). |
|  | 18b | Responsibility of study coordinator: Pag 10 of manuscript |
| Data management | 19 | Pag 9 of manuscript |
| Statistical methods | 20a | Pag 9 of manuscript |
|  | 20b | Methods for any additional analyses: no applicable in this trial |
|  | 20c | Definition of analysis population relating to protocol non-adherence (eg, as randomised analysis), and any statistical methods to handle missing data (eg, multiple imputation): no applicable in this trial |
| **Methods: Monitoring** | | |
| Data monitoring | 21a | Pag 8 -9 of manuscript |
|  | 21b | Description of any interim analyses and stopping guidelines, including who will have access to these interim results and make the final decision to terminate the trial: Not applicable. There is no interim analysis or stopping rules in this trial. |
| Harms | 22 | Pag 10 of the manuscript |
| Auditing | 23 | Not applicable. There is no on-site auditing of the trial. Before exporting the data into a computerized database at the data coordinating center, local investigators will check the completeness and the quality of information. |
| Ethics and dissemination | | |
| Research ethics approval | 24 | Pag 5-12 of the manuscript |
| Protocol amendments | 25 | The Steering Committee will communicate substantial protocol modifications (when applicable) to relevant parties (investigators, Ethics Committee). |
| Consent or assent | 26a | Pag 5-12 of the manuscript (and Additional file 2) |
|  | 26b | Additional consent provisions for collection and use of participant data and biological specimens in ancillary studies. No applicable |
| Confidentiality | 27 | Patient information is anonymized |
| Declaration of interests | 28 | Pag 13 of the manuscript |
| Access to data | 29 | Pag 13 of the manuscript |
| Ancillary and post-trial care | 30 | No procedure will be used outside of normal practice. Therefore the trial will be covered by the National Health Service in Spain. |
| Dissemination policy | 31a | Results of the trial will be published in international journals. There is no obligation for communicating the results to individual patients. |
|  | 31b | All participant investigators have the right to be authors of the final publication. |
|  | 31c | Not applicable |
| Appendices |  |  |
| Informed consent materials | 32 | The model of the informed consent is provided as the Additional File 2. |
| Biological specimens | 33 | Not applicable |

*It is strongly recommended that this checklist be read in conjunction with the SPIRIT 2013 Explanation & Elaboration for important clarification on the items. Amendments to the protocol should be tracked and dated. The SPIRIT checklist is copyrighted by the SPIRIT Group under the Creative Commons “[Attribution-NonCommercial-NoDerivs 3.0 Unported](http://www.creativecommons.org/licenses/by-nc-nd/3.0/)” license.
